# Letter from Elgin

**Published:** 1882-11

**Authors:** H. Rosencrans

**Affiliations:** Elgin, Ill.


### Article XIV.

Elgin, Ill., October 7, 1882.

Editors Medical Journal and Examiner:

A Mrs. C., æt. thirty years; mother of two living children, a boy of seven, and girl of five years. This patient was treated by me during the years 1877-8, for abdominal dropsy. She was very large when first seen by me. She stated to me that her former physician first located the tumor in the left hypogastric region. That was two years previous to my first seeing her. She was considerably emaciated, and vomited a great deal. I performed paracentesis abdominis, an inch and a half below the umbilicus; drew off about three gallons of fluid. During my treatment of about two years, when she died, I tapped her six times. drawing off about 150 lbs. of fluid. Every tapping relieved her for awhile.

*Autopsy* revealed hundreds of cysts in the peritoneal sac, of all colors and sizes (the largest about the size of a medium-sized orange), in clusters like grapes; the contents of these cysts were very gelatinous. During the operation, many fell on the floor. So slippery was this stuff, that it was very difficult to grasp it in the hand. It would slip out and shake like jelly. What were these cysts? Were they *echinococis hominis?* I never saw the like before, nor since. I had to assist me in the post-mortem Dr. Fisher, of Matagordas, Texas. This sight to us was very interesting and marvelous. Some time has elapsed since that, but I have often thought of it, and wished that I was better enlightened on the subject of what constituted and what caused these hundreds of cysts of all sizes and all colors. There did seem to be a connection with the left ovary. The uterus was healthy. It may be that this condition of things is common in peritoneal dropsy, but I had treated a number of cases, but never before witnessed it. H. ROSENCRANS, M.D.

---

THE SOCIETY OF PHYSICIANS AND SURGEONS OF MINNEAPOLIS.—A medical society under the above title was organized October 15, by the regular physicians of Minneapolis. Its corps of officers for the ensuing year is as follows:

President, Dr. O. S. Chapman; Vice-President, Dr. S. Keith; Secretary, Dr. Charles W. Drew; Treasurer, Dr. E. J. Brown; Board of Censors, Dr. S. F. Hance, Dr. A. B. Cates, Dr. W. H. Byford, Jr.; Committee on Ethics, Dr. Geo. F. French, Dr. F. E. Towers, Dr. W. S. Laton; Committee on Entertainment, Dr. A. H. Hedderly, Dr. J. E. Moore, Dr. H. N. Orton. Success to them. There are also significant signs of the early foundation of a college of pharmacy in Minneapolis.

ANTISEPTIC COLOGNE.—

℞ Eau de cologne..............................℥viij.

Chloral hydrate..............................ʒij.

Quinine..............................gr. x.

Carbolic acid (pure)..............................gr. xxx.

Oil of lavender..............................gtt xx. M.

